# Collagen Sponge Functionalized with Chimeric Anti-BMP-2 Monoclonal Antibody Mediates Repair of Critical-Size Mandibular Continuity Defects in a Nonhuman Primate Model

**DOI:** 10.1155/2017/8094152

**Published:** 2017-03-16

**Authors:** Yilin Xie, Yingying Su, Seiko Min, Jianxia Tang, Bee Tin Goh, Leonardo Saigo, Sahar Ansari, Alireza Moshaverinia, Chunmei Zhang, Jinsong Wang, Yi Liu, Arash Khojasteh, Homayoun H. Zadeh, Songlin Wang

**Affiliations:** ^1^Molecular Laboratory for Gene Therapy and Tooth Regeneration, Beijing Key Laboratory of Tooth Regeneration and Function Reconstruction, Capital Medical University School of Stomatology, Tian Tan Xi Li No. 4, Beijing 100050, China; ^2^Laboratory of Tissue Regeneration and Immunology and Department of Periodontics, Beijing Key Laboratory of Tooth Regeneration and Function Reconstruction, Capital Medical University School of Stomatology, Tian Tan Xi Li No. 4, Beijing 100050, China; ^3^Laboratory for Immunoregulation and Tissue Engineering (LITE), Ostrow School of Dentistry, University of Southern California, Los Angeles, CA, USA; ^4^Department of Oral and Maxillofacial Surgery, Xiangya Stomatological Hospital, Central South University, Changsha, Hunan, China; ^5^Department of Oral & Maxillofacial Surgery, National Dental Centre, Singapore; ^6^Division of Growth and Development, School of Dentistry, University of California, Los Angeles, CA, USA; ^7^Division of Advanced Prosthodontics, School of Dentistry, University of California, Los Angeles, CA, USA; ^8^Department of Biochemistry and Molecular Biology, Capital Medical University School of Basic Medical Sciences, You An Men Wai Xi Tou Tiao No. 10, Beijing 100069, China; ^9^Department of Tissue Engineering, School of Advanced Technologies in Medicine, Shahid Beheshti University of Medical Sciences, Tehran, Iran

## Abstract

Antibody-mediated osseous regeneration (AMOR) has been introduced by our research group as a tissue engineering approach to capture of endogenous growth factors through the application of specific monoclonal antibodies (mAbs) immobilized on a scaffold. Specifically, anti-Bone Morphogenetic Protein- (BMP-) 2 mAbs have been demonstrated to be efficacious in mediating bone repair in a number of bone defects. The present study sought to investigate the application of AMOR for repair of mandibular continuity defect in nonhuman primates. Critical-sized mandibular continuity defects were created in* Macaca fascicularis* locally implanted with absorbable collagen sponges (ACS) functionalized with chimeric anti-BMP-2 mAb or isotype control mAb. 2D and 3D analysis of cone beam computed tomography (CBCT) imaging demonstrated increased bone density and volume observed within mandibular continuity defects implanted with collagen scaffolds functionalized with anti-BMP-2 mAb, compared with isotype-matched control mAb. Both CBCT imaging and histologic examination demonstrated de novo bone formation that was in direct apposition to the margins of the resected bone. It is hypothesized that bone injury may be necessary for AMOR. This is evidenced by de novo bone formation adjacent to resected bone margins, which may be the source of endogenous BMPs captured by anti-BMP-2 mAb, in turn mediating bone repair.

## 1. Introduction

Loss of mandibular bone due to congenital anomalies, trauma, infection, or tumor resection surgeries is a challenging clinical problem for reconstruction. Current methods for repair or regeneration include autologous bone grafting, allogenic bone grafting, and tissue engineering [1–3]. For several decades, the most widely used procedures to promote healing of bone fractures and large defects utilized autologous or allogenic bone grafts [1]. However, the use of these materials has a number of drawbacks including potential host reaction, limited donor tissue availability, donor-site morbidity, and potential disease transmission from allografts [4]. An alternative to bone grafts is bone tissue engineering. Tissue engineering entails the application of progenitor cells and/or growth factors delivered to the treatment site on an acellular scaffold. It is well known that bone tissue engineering is partially regulated by the host local microenvironment, including the presence of signaling molecules and host immune cells [5–7]. Bone Morphogenetic Proteins (BMPs) are potential osteoinductive growth factors that play a critical role in bone regeneration and repair [8]. It is well known that exogenous administration of recombinant human (rh) BMP-2 can initiate a healing cascade that mediates bone regeneration through the TGF-*β*/BMP signaling pathway [8]. Owing to their substantial osteogenic properties, the US Food and Drug Administration (FDA) had approved rhBMP-2 and rhBMP-7 for clinical use [9, 10]. However, application of exogenous growth factors also has a number of drawbacks, including serious adverse effects which are occasionally fatal; moreover, recombinant growth factors have reduced biological activity, requiring high concentrations to be used in vivo and therefore are associated with high costs [5, 11–13]. These shortcomings have driven the quest for the development of alternative strategies. Our research group has introduced an alternative bone tissue engineering approach termed antibody-mediated osseous regeneration (AMOR). We have identified specific monoclonal antibody (mAb) clones directed against BMP-2 that have the ability to capture endogenous BMP-2 ligands and present them to progenitor cells, mediating in vivo tissue repair [14]. Ansari et al. reported that AMOR can be regulated through BMP signaling pathway [15]. In addition, it was confirmed that anti-BMP-2 mAb can trap and tether endogenous BMP-2 ligands for the directed osteodifferentiation of mesenchymal stem cells through in vitro and in vivo studies [16]. In previous studies, murine and chimeric anti-BMP-2 mAb has been used in mice, rat, and rabbit models and confirmed to be effective in promoting bone repair and regeneration through calvaria defect models [17–20]. However, there has been a need for more clinically relevant animal models. Specifically, more relevant animal models for craniofacial reconstructive surgeries are desired. Before a novel approach like this one can be introduced into a clinical trial, preclinical studies should be available in larger animal models that more closely mimic challenging human clinical skeletal defects. Therefore, in the present study, we investigated the ability of chimeric anti-BMP-2 mAb to mediate repair of a critical-size mandibular continuity defect in a nonhuman primate model.

## 2. Materials and Methods

### 2.1. Animals

All animal experiments were performed in accordance with a protocol approved by the Institutional Animal Care and Use Committee (IACUC) of the Capital Medical University, Beijing, China. Six adult male crab-eating macaques* (Macaca fascicularis)* aged 8–12 and weighing between 4.0 and 5.0 kg were included in this study. Three animals were assigned to experimental (AMOR) and 3 to control (isotype-matched mAb) groups. Before surgery, the animals were housed in individual cages with water and fed ad libitum.

### 2.2. Antibody

The hybridoma clone of a murine anti-BMP-2 mAb was expanded and used in order to generate chimeric anti-BMP-2 mAb according to procedures described by Ansari et al. [15]. Based on previous dose-response data, 25 *μ*g/mL was selected as the optimal dose of chimeric anti-BMP-2 mAb for all experiments [15, 18].

### 2.3. Scaffold Biomaterials

Chimeric anti-BMP-2 mAb (25 *μ*g/mL) and isotype control mAb (25 *μ*g/mL) were adsorbed on type 1 absorbable collagen sponge (ACS; CollaCote: Integra, Plainsboro, NJ, USA) as previously described [17]. Briefly, ACS was saturated in diluted mAb for one hour at room temperature prior to in vivo implantation.

### 2.4. Mandibular Defect Model

**Figure 1:**
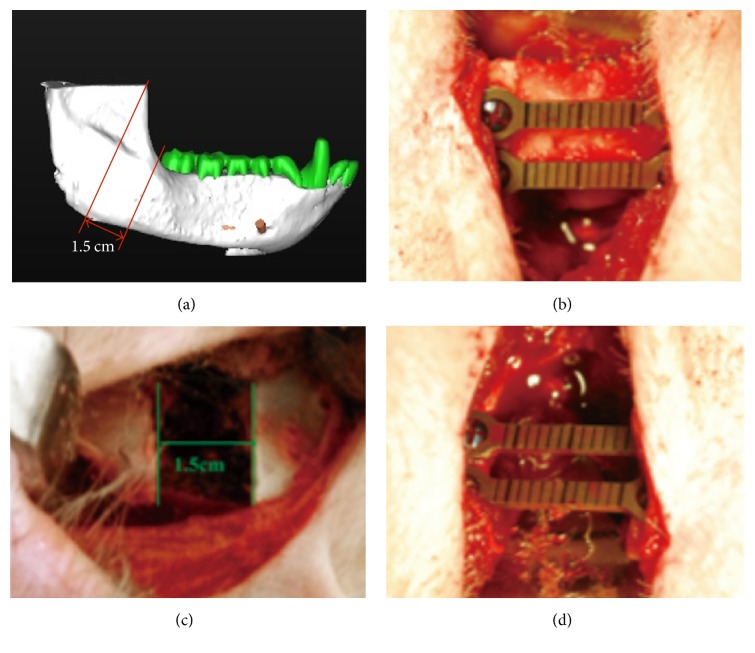
Representative clinical and 3D-rendered imaging volume of mandibular continuity defects. Preoperative CBCT images (a) were used to aid in planning of the segmental osteotomy, as well as in comparison with postsurgical images. Clinical view, showing the surgically exposed posterior mandible, where the reconstruction plates were provisionally attached to the mandible (b). This step defined the position of the titanium screws and reconstruction plates relative to the planned osteotomy to aid in orienting the segmented ends following resection back in correct position. Resected segmental osteotomy, creating a 1.5 cm continuity defect (c). Repositioned mandibular segments fixated with titanium reconstruction plates (d).

Six animals were randomly assigned into experimental or control groups. The three animals in the control group received ACS immobilized with isotype-matched control mAb and the three experimental animals received ACS immobilized with chimeric anti-BMP-2 mAb in their defects. Animals were sedated with subcutaneous injection of 5 mg/kg of ketamine (Jiangsu Hengrui Medicine Co., Ltd, Lianyungang, China). Anesthesia was achieved by veterinarian staff with IV Propofol (8 mg/kg; Diprivan, Astra Zeneca, London, England). Endotracheal intubation was performed using an oral-tracheal tube with a diameter of 3.5 mm (Sheridan™, Teleflex Medical, NC, USA). The anesthesia was maintained by IV Propofol (2.5 mg/kg) per 30~45 min. Local anesthesia was achieved by intramucosal injection of lidocaine with 1 : 100,000 epinephrine (Astra Zeneca, London, England) [21, 22]. Hair over the right mandibular region was shaved. The animals were swabbed periorally with 1% Cetrimide followed by 0.05% chlorhexidine gluconate solution. All surgical sites were washed with 0.12% chlorhexidine gluconate solution. In each animal, a 4 cm submandibular incision was made at 1 cm below the inferior border of the mandible, after which the platysma was identified and cut, taking care not to damage the mandibular branch of the facial nerve. The facial artery and vein were then identified, tied off, and ligated. The masseter muscle was then identified and dissected to reach the periosteal layer. The periosteum was incised and elevated to expose the mandible to the region of the first molar anteriorly and halfway up the ascending ramus posteriorly. Preoperative CBCT images were taken in order to aid with planning the segmental osteotomies (Figure 1(a)). Reconstruction plates were provisionally attached to the mandible over the region planned for resection, prior to osteotomy (Figure 1(b)). This step defined the position of the titanium screws and reconstruction plates relative to the planned osteotomy to help with orienting the segmented ends, following resection back in correct position. A 1.5 cm wide segmental osteotomy of the mandible was performed on the right angle of mandible, using a rotary fissure bur under copious irrigation (Figure 1(c)). The mandibular segments were reoriented using the positions of the predrilled titanium screws as a guide. The segments were fixated with 2 titanium reconstruction plates using two titanium screws per plate (Figure 1(d)). ACS scaffold (4.0 cm × 2.0 cm × 3.0 mm), which was preincubated with anti-BMP-2 or isotype-matched control mAb, was placed within experimental or control transected defect sites, respectively. Since the scaffolds were spongy in consistency, once incubated with 100 *μ*L of anti-BMP-2 or isotype control mAb, they easily adapted to the confines of the defects. The incision was approximated primarily in layers using polyglactin sutures (Vicryl™, Ethicon Inc, Somerville, NJ, USA). The skin was covered with 3% tetracycline hydrochloride antibiotic ointment. Postoperative care included analgesia by IM injection of Carprofen (2 mg/kg; Harbin Pharmaceutical Group, Co., Ltd, Harbin, China) for 3 days postoperatively. Animals were maintained on a soft diet for 2 weeks postoperatively. Oral hygiene measure consisted of spraying the teeth of animals with 0.2% chlorhexidine gluconate mouthwash (Etouch, Shandong, China) once a day.

### 2.5. Cone Beam Computed Tomography (CBCT) Image Analysis

Live animals were imaged with CBCT scanner (Kodak Medical Solutions Molecular Imaging, Knoxville, TN) with 60 *μ*m voxel size at 60 kV and 110 mA at three time points (preoperatively, as well as at 6 and 12 weeks postoperatively). The data were acquired in Digital Imaging and Communications in Medicine (DICOM) format. For two-dimensional analysis, DICOM files were imported into Simplant Pro 16.0 Software (Dentsply Implants, Waltham, MA, USA) for 2D and 3D image reconstruction and density analysis. The minimum threshold used for reconstruction of 3D volumes was set at 400 Hounsfield units (HU), based on the lowest density observed within control and experimental specimens. This threshold was used for all pre- and postoperative images at all time points. Pre- and postoperative (6 and 12 weeks after surgery) CBCT images were examined for 2D quantitative analysis of de novo bone formation by density measurements within sections taken in axial planes. For analysis of axial images, 3 sections were taken at equal distances from the superior-most through inferior-most boundaries of the defects. The density measurements were performed, using rectangle density measurement tool in Simplant software in each of the sections, and the density was recorded in HU. Titanium plates were excluded from analysis of density.

3D image analysis was performed as previously described [22]. Briefly, DICOM files were imported into Mimics software (Materialise, Leuven, Belgium) to construct 3D volumes. The reconstructions of scanned images were thresholded to remove any soft tissue and cartilage, leaving only mineralized tissues, and sectioned to isolate the defect site. The 3D reconstructed volumes were exported in Stereolithography (STL) file format and imported into reverse engineering software (Geomagic Studio® v12.0 software, 3D SYSTEMS, Cary, NC, USA). Since file sizes were very large, slowing down analysis, once volumes were reconstructed, images were then trimmed in order to facilitate manipulations. Landmarks to define the boundaries of surgical defects were selected as 15 mm, based on titanium plates used as reference. The volumes present within confines of defects at 6 and 12 week postsurgical time points were quantified and expressed in mm^3^.

### 2.6. Histology and Histomorphometry

Twelve weeks after surgical procedure, the animals were euthanized. The specimens were fixed with 4% (v/v) paraformaldehyde for 24 hours at room temperature. Samples were then decalcified in ethylene diamine tetraacetic acid (EDTA) for 60 days. The samples were dehydrated in a graded ethanol series (70%, 95%, and 100%) and embedded in paraffin. The specimens were then serially sectioned (10 *μ*m), deparaffinized, hydrated, and stained with Hematoxylin and Eosin (H&E). The stained sections were viewed under microscope (Olympus, Tokyo, Japan) at various magnifications and digital images were acquired. Quantitative histomorphometry was performed, using NIH Image J software (U.S. National Institutes of Health, Bethesda, Maryland, USA). Images taken at 40x were used for histomorphometry. Standardized nomenclature was used for defining various components of tissues within each viewed field [23]. Newly formed bone volume (BV) is defined as regions containing osteocytes within lacunae, the proportion of which was calculated relative to that of total tissue volume (TV).

### 2.7. Statistical Analysis of Data

Quantitative data were presented as mean ± standard deviation (SD). Comparisons between two groups were performed using two-tailed unpaired Student's* t*-tests. Differences were considered statistically significant at a *P* value < 0.05.

## 3. Results

### 3.1. Clinical Outcomes

All animals healed uneventfully without any adverse biologic complications. All surgical sites showed minimal inflammation and no signs of infection. Animals were euthanized at 12 weeks postsurgically.

### 3.2. Analysis of Mineralized Tissue Formation by CBCT

**Figure 2:**
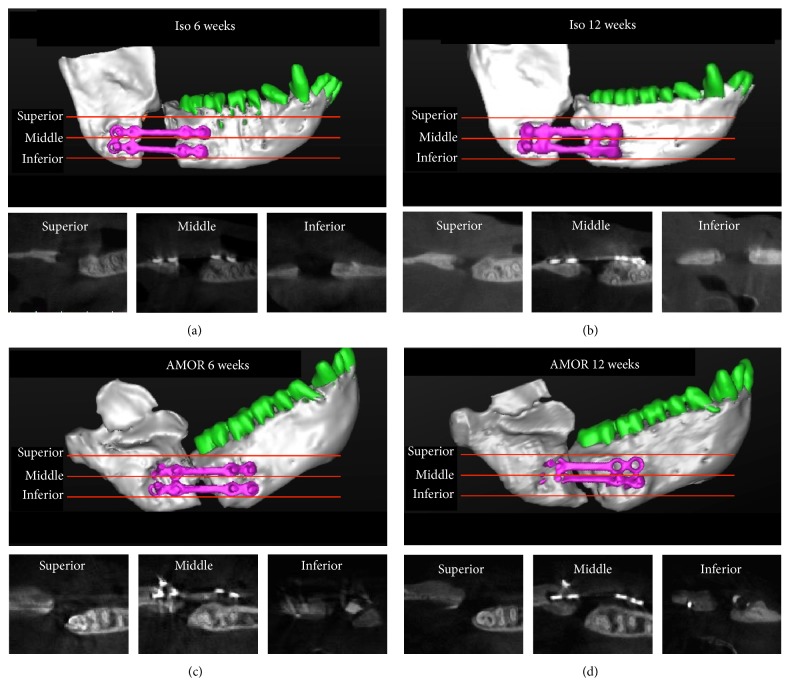
Representative 2D and 3D reconstructed CBCT images of mandibular continuity defects of experimental and control groups at 6 and 12 weeks postsurgically. 3D reconstructed CBCT images illustrate the landmarks used for axial plane sections for 2D image analysis. Accordingly, axial sections were obtained at equal distances from the superior region of the mandibular defects, above titanium plates (superior plane), as well as at middle (middle plane) and inferior regions (inferior plane) of the mandible. Representative 2D and 3D reconstructed images of defects treated with isotype-matched control mAb (a, b) or chimeric anti-BMP-2 mAb (c, d) at 6 (a, c) and 12 (b, d) time points are illustrated.

To investigate the ability of the mAb to repair large critical-size craniofacial defects, 15 mm continuity defects were surgically created in the posterior mandible and the two segments were rigidly fixated with titanium reconstruction plates (Figure 1). The 15 mm defect was filled with collagen scaffold functionalized with chimeric anti-BMP-2 mAb or isotype-matched control mAb. The areas were allowed to heal for 12 weeks. To investigate the kinetics of bone healing, serial CBCT imaging was conducted preoperatively as well as 6 and 12 weeks postoperatively. The CBCT images (Figure 2) were subjected to 2D and 3D quantitative analysis to determine the degree of bone healing within experimental and control defects at the two postoperative time points. The 2D CBCT images were examined in three discrete planes, that is, coronal, axial, and sagittal. Moreover, three different zones within each of the axial planes (superior, middle, and inferior) (Figure 2) were analyzed. Regions of interest were defined within coronal (anterior, middle, and posterior) and sagittal (anterior, middle, and posterior) planes (Supplemental Figure 1 in Supplementary Material available online at https://doi.org/10.1155/2017/8094152). The bone density within those defined regions is reported in Supplemental Table 1.

**Figure 3:**
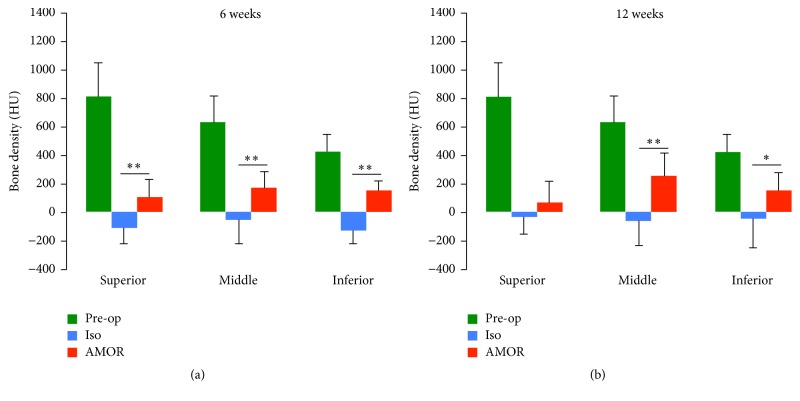
Quantitative analysis of bone density within axial images of CBCTs taken presurgically and at 6 (a) and 12 (b) weeks postsurgically. Bone density was measured within axial sections of CBCT images obtained from superior, middle, and inferior regions, according to the landmarks illustrated in Figure 2. The mean density of experimental mandibular area at preoperative time point is presented for comparison. Significantly higher bone density was observed within defects treated with chimeric anti-BMP-2 mAb compared to isotype-matched control mAb (^*∗*^*P* < 0.05, ^*∗∗*^*P* < 0.01).

Results demonstrated that mandibular defects implanted with isotype-matched control mAb immobilized on collagen scaffold exhibited very low density with negative HU at 6 and 12 weeks postoperatively (Figure 3). In contrast, mandibular defects implanted with chimeric anti-BMP-2 mAb immobilized on collagen scaffold exhibited significantly higher density consistent with formation of mineralized tissue after 6 weeks, which increased by 12 weeks (Figure 3). Qualitative examination of 2D and 3D reconstructed images illustrated that new bone formation was observed at the margins of the defect adjacent to resected parent bone.

**Figure 4:**
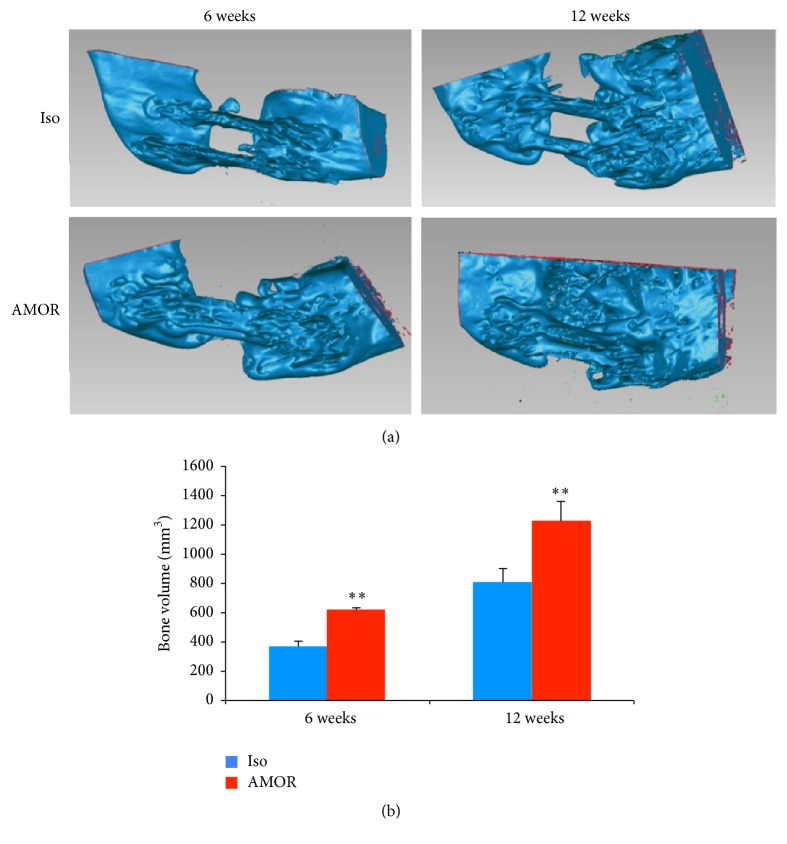
3D analysis of bone volume within surgical defect. Mandibular continuity defects were treated with chimeric anti-BMP-2 mAb compared to isotype-matched control mAb. CBCT images taken at 6 and 12 weeks postsurgically and the volume of bone within the defect sites at two time points was quantified (a). Quantitative analysis of bone volume (mm^3^) within defects treated with chimeric anti-BMP-2 mAb or isotype-matched control mAb (b). Significantly higher bone volume was detected within defects treated with chimeric anti-BMP-2 mAb compared to isotype-matched control mAb at both time points (^*∗∗*^*P* < 0.01).

Next, 3D volumetric measurement was performed for tissues formed within surgical defects at 6 and 12 weeks postoperatively (Figure 4(a)). Results revealed increasing bone volume from 6 to 12 weeks in both experimentally treated or control-treated defects. At both 6 and 12 weeks, there was significantly more mineralized tissue volume, within defects treated with chimeric anti-BMP-2 mAb (Figure 4(b)).

### 3.3. Histologic and Histomorphometric Analysis

**Figure 5:**
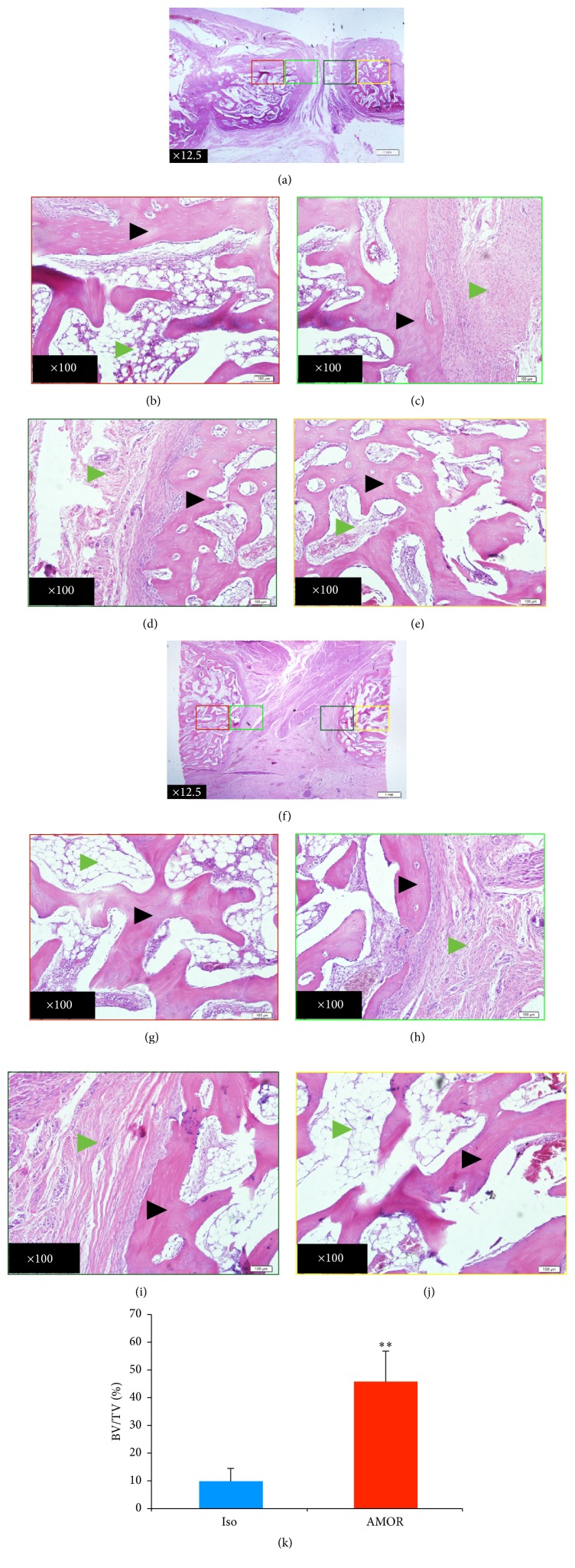
Histologic and histomorphometric analysis of treated mandibular defects. Histomicrographs of H&E stained sections obtained from axial planes taken at the midpoint between superior and inferior boundaries of mandibular defects (a–j). Mandibular segmental osteotomy defects were treated with chimeric anti-BMP-2 mAb (a–e) or isotype-matched control mAb (f–j) immobilized on absorbable collagen sponge scaffold. Histological evaluation 12 weeks after surgery revealed the presence of mature lamellar bone in the newly ossified tissue of experimental defect treated with chimeric anti-BMP-2 mAb (a–e), whereas isotype-matched control mAb (f–j) was primarily filled with connective tissue. There was no evidence of inflammatory infiltration in either group. Black and green arrowheads represent osteoid bone or all other tissues (referred to as void), respectively. Quantitative histomorphometric analysis of new bone formation (k) confirmed higher degree of bone formation within defects treated with chimeric anti-BMP-2 mAb compared with isotype-matched control mAb (^*∗∗*^*P* < 0.01).

To examine the nature of biologic healing at the cellular level, qualitative histological examination was performed on the treated mandibles at 12 weeks postoperatively (Figure 5). Histological evaluation revealed the presence of mature lamellar bone containing osteocytes within lacunae within newly ossified tissues in defects implanted with ACS functionalized with anti-BMP-2 mAb (Figures 5(a)–5(e)). On the other hand, defects implanted with ACS and isotype-matched control mAb were occupied primarily by connective tissue (Figures 5(f)–5(j)). Similar to imaging data, histomicrographs illustrated that new bone formation was observed in direct apposition to the margins of old bone. There was no evidence of local inflammatory infiltration in either group. Quantitative histomorphometric analysis revealed greater percentage of BV/TV within defects implanted with ACS functionalized with anti-BMP-2 mAb compared with isotype-matched control mAb (Figure 5(k)).

## 4. Discussion

Autogenous bone grafting has long been regarded as the gold standard for bone augmentation. However, there are many disadvantages associated with this treatment modality such as infection, hematoma, morbidity, and increased surgical expense. The use of allografts, xenografts, and synthetic biomaterials has enabled clinicians to avoid some of the disadvantages of autografts; however, application of these types of materials is still limited. As an alternative treatment option, involving application of rhBMP-2 has been widely used for bone regenerative procedures, providing a promising alternative therapeutic option to autologous bone grafting. However, there are several disadvantages associated with the application of rhBMP-2. Recent studies from our research group have demonstrated a tissue engineering strategy involving immobilization of anti-BMP-2 mAb on various scaffolds implanted into surgical defect sites [14, 15, 17–20]. AMOR has been validated in calvarial defects of rats and rabbits [14, 15, 17–20]. In order to advance this translational project toward our ultimate goal of clinical trial, the present investigation sought to utilize a clinically relevant defect in an animal model that is phylogenetically closer to human. In the present study, critical-size continuity mandibular defects were generated in a nonhuman primate model. This is a clinically relevant animal model, mimicking many conditions where craniofacial bones are resected due to neoplastic, traumatic, or inflammatory lesions. Using this animal model, which is closely associated with human, we demonstrated the capacity of collagen scaffold functionalized with anti-BMP-2 mAb to mediate de novo bone formation within mandibular continuity defects. These data confirmed and extended our previous studies, demonstrating the efficacy of AMOR in rodents and rabbits [17–20].

Before a tissue engineering approach can be critically examined in a clinical trial, confirming the findings in a large animal model that closely mimics the clinical condition being studied is required [24, 25]. Our results provide further evidence in support of AMOR as a translational approach. Its efficacy and reproducibility have been demonstrated in a number of animals with different defect models [14–20].

The present experimental model is the largest defect which we have utilized to investigate the mechanism of AMOR. The present model has provided significant insights into the mechanism of osteogenesis within these defect sites. Firstly, the fact that application of anti-BMP-2 mAbs that were generated in a murine host against human BMP-2 antigen led to repair of large critical-size defects in monkeys suggests that this antibody is capable of capturing endogenous monkey BMP ligands. This may be attributed to the high degree of homology between human and monkey BMP molecules [26]. Secondly, two possible mechanisms may be suggested for osteogenesis through AMOR: (1) osteoconduction, that is, bone formation by direct opposition on parent bone, and (2) de novo bone formation, which is bone repair without existing bone template. The present experiment showed bone repair was initiated and propagated at the margins of surgical defects. It may be hypothesized that the concentrations of endogenous BMPs are greatest at host bone that is responsible for this pattern of bone formation.

Over the past three decades, monoclonal antibody therapy has become one of the fastest growing areas of biopharmaceutical applications. The reason for the increasing popularity of therapeutic mAbs is their safety profile, high degree of specificity, diversity, and relatively low cost. The majority of current therapeutic mAbs are utilized for targeted therapy in three main areas, namely, cancer, immunity, and inflammation [27]. To date, the primary route of antibody administration is systemic administration, which may cause “infusion reaction” [27]. In the present study, anti-BMP-2 mAb was delivered using a collagen scaffold and implanted locally into the defect sites to mediate de novo bone formation. We have previously demonstrated that the locally implanted anti-BMP-2 mAb is slowly released, though it persists for at least 8 weeks [15, 19]. There were no side effects or adverse events detected in the current study. This is consistent with previous studies conducted in other animal models [17–20], suggesting the safety of local administration of anti-BMP-2 mAbs.

It has been demonstrated that BMPs in solution are quickly cleared from the body, which may explain why high doses of exogenous rhBMPs are needed for tissue regeneration [16]. The high dose of rhBMP currently approved by the FDA (1.5 mg/mL) is several orders of magnitude above physiological levels. Significant complications have been reported following rhBMP-2 administration, perhaps due to the superphysiologic concentration [28]. In the present study, we used ACS as the scaffold for immobilizing anti-BMP-2 mAb and observed significant de novo bone formation in large critical-size mandibular continuity defects. The contrasts between the application of exogenous growth factors and in situ trapping of endogenous growth factors by specific monoclonal antibodies are multifold:

1. Spatial and temporal availability of growth factors is linked to the biological process of wound healing. In normal biological wound healing, growth factors need to be present at the appropriate concentration in the local microenvironment at the appropriate time. In some circumstances, the availability of growth factors at an inappropriate time can have the opposite of the intended effect.
2. Endogenous growth factors have higher biological activity than their recombinant counterparts, presumably due to differences in posttranslational modification of endogenous growth factors.
3. Antibodies have excellent safety profiles, particularly when delivered locally as in AMOR.

In AMOR, the availability of growth factors is orchestrated by endogenous expression of those growth factors, expressed at the appropriate stage in the cascade of events during wound healing. AMOR merely amplifies the effects of the growth factors by facilitating their local accumulation via specific mAbs.

Since antibody therapy has been associated with some potential adverse reactions [29], it was important to address this issue. We carefully examined our histologic sections of mandible for presence of any signs of adverse reaction such as exuberant inflammatory infiltrate and we did not observe significant inflammatory infiltrate in experimental or control sites. This is most likely due to the fact that the volume of diluted mAb solution (150 *μ*L) at 25 *μ*g/mL concentration used for incubation with the scaffolds entails maximum of 3.75 *μ*g of mAb administered. This dose is far lower than those used for typical therapeutic mAbs that are clinically administered. Moreover, since AMOR entails applying mAbs, which are immobilized on solid scaffold, the potential for adverse reaction is likely lower than most therapeutic mAbs which are systemically administered.

In view of the many complications and limitations reported for the current gold standard of bone repair (e.g., autogenous grafting) and the newer technology of recombinant growth factor therapy, there is currently a need for safe and effective alternatives for the treatment of significant skeletal defects. AMOR presents a viable alternative strategy and merits further investigation. Although chimeric antibodies are currently approved by the US FDA for clinical use, most of the therapeutic antibodies currently in use are humanized or fully human. Humanized antibodies are generated by transfer of the complementarity determining region (CDR) of the antibody from another species onto a human Ab. Therefore, before clinical studies can be conducted on AMOR, humanized antibodies have to be generated. Currently, generation of humanized anti-BMP-2 mAbs which are suitable for clinical testing is in the planning stages.

## 5. Conclusion

In the present study, chimeric anti-BMP-2 mAb immobilized on ACS scaffold promoted de novo bone formation efficiently, as confirmed by a novel clinically relevant mandibular continuity defect in a nonhuman primate model. Altogether, our data demonstrate the efficacy of chimeric anti-BMP-2 mAb for bone tissue engineering applications. The strategy proposed herein has a multitude of potential clinical applications in the repair of skeletal defects due to congenital, traumatic, neoplastic, or inflammatory processes.

## Supplementary Material

Supplemental Figure 1: 3D reconstructed CBCT images of a mandibular continuity defect at preoperative time point. 3D reconstructed images illustrate the landmarks used for 2D image analysis. Coronal (A) and sagittal (B) sections were obtained at equal distances from the anterior region to posterior region of defect as well as distance from buccal to lingual region of defect respectively.
Supplemental Table 1: The bone density (HU) at three different zone within Axial, Coronal, and Sagittal places of CBCTs taken at 6 and 12 weeks.

## References

[1] Giannoudis P. V., Dinopoulos H., Tsiridis E. (2005). Bone substitutes: an update. *Injury*.

[2] Monaco E., Bionaz M., Hollister S. J., Wheeler M. B. (2011). Strategies for regeneration of the bone using porcine adult adipose-derived mesenchymal stem cells. *Theriogenology*.

[3] Betz V. M., Betz O. B., Harris M. B., Vrahas M. S., Evans C. H. (2008). Bone tissue engineering and repair by gene therapy. *Frontiers in Bioscience*.

[4] De Long W. G., Einhorn T. A., Koval K. (2007). Bone grafts and bone graft substitutes in orthopaedic trauma surgery. A critical analysis. *The Journal of Bone & Joint Surgery—American Volume*.

[5] Chin M., Ng T., Tom W. K., Carstens M. (2005). Repair of alveolar clefts with recombinant human bone morphogenetic protein (rhBMP-2) in patients with clefts. *Journal of Craniofacial Surgery*.

[6] Kratchmarova I., Blagoev B., Haack-Sorensen M., Kassem M., Mann M. (2005). Cell signalling: mechanism of divergent growth factor effects in mesenchymal stem cell differentiation. *Science*.

[7] Czyz J., Wobus A. M. (2001). Embryonic stem cell differentiation: the role of extracellular factors. *Differentiation*.

[8] Groeneveld E. H. J., Burger E. H. (2000). Bone morphogenetic proteins in human bone regeneration. *European Journal of Endocrinology*.

[9] Hsu W. K., Wang J. C. (2008). The use of bone morphogenetic protein in spine fusion. *Spine Journal*.

[10] Jones A. L., Bucholz R. W., Bosse M. J. (2006). Recombinant human BMP-2 and allograft compared with autogenous bone graft for reconstruction of diaphyseal tibial fractures with cortical defects. A randomized, controlled trial. *The Journal of Bone & Joint Surgery—American Volume*.

[11] Khan S. N., Lane J. M. (2004). The use of recombinant human bone morphogenetic protein-2 (rhBMP-2) in orthopaedic applications. *Expert Opinion on Biological Therapy*.

[12] Zhu W., Rawlins B. A., Boachie-Adjei O. (2004). Combined bone morphogenetic protein-2 and -7 gene transfer enhances osteoblastic differentiation and spine fusion in a rodent model. *Journal of Bone and Mineral Research*.

[13] Wikesjö U. M. E., Polimeni G., Qahash M. (2005). Tissue engineering with recombinant human bone morphogenetic protein-2 for alveolar augmentation and oral implant osseointegration: experimental observations and clinical perspectives. *Clinical Implant Dentistry and Related Research*.

[14] Ansari S., Freire M., Choi M. G. (2015). Effects of the orientation of anti-BMP2 monoclonal antibody immobilized on scaffold in antibody-mediated osseous regeneration. *Journal of Biomaterials Applications*.

[15] Ansari S., Moshaverinia A., Pi S. H., Han A., Abdelhamid A. I., Zadeh H. H. (2013). Functionalization of scaffolds with chimeric anti-BMP-2 monoclonal antibodies for osseous regeneration. *Biomaterials*.

[16] Moshaverinia A., Ansari S., Chen C. (2013). Co-encapsulation of anti-BMP2 monoclonal antibody and mesenchymal stem cells in alginate microspheres for bone tissue engineering. *Biomaterials*.

[17] Freire M. O., You H.-K., Kook J.-K., Choi J.-H., Zadeh H. H. (2011). Antibody-mediated osseous regeneration: a novel strategy for bioengineering bone by immobilized anti-bone morphogenetic protein-2 antibodies. *Tissue Engineering Part A*.

[18] Freire M. O., Kim H. K., Kook J.-K., Nguyen A., Zadeh H. H. (2013). Antibody-mediated osseous regeneration: the early events in the healing response. *Tissue Engineering. Part A*.

[19] Ansari S., Freire M. O., Pang E.-K., Abdelhamid A. I., Almohaimeed M., Zadeh H. H. (2014). Immobilization of murine anti-BMP-2 monoclonal antibody on various biomaterials for bone tissue engineering. *BioMed Research International*.

[20] Freire M., Choi J.-H., Nguyen A. (2015). Application of AMOR in craniofacial rabbit bone bioengineering. *BioMed Research International*.

[21] Min S., Liu Y., Tang J. (2016). Alveolar ridge dimensional changes following ridge preservation procedure with novel devices: part 1—CBCT linear analysis in non-human primate model. *Clinical Oral Implants Research*.

[22] Omran M., Min S., Abdelhamid A., Liu Y., Zadeh H. H. (2016). Alveolar ridge dimensional changes following ridge preservation procedure: part-2—CBCT 3D analysis in non-human primate model. *Clinical Oral Implants Research*.

[23] Dempster D. W., Compston J. E., Drezner M. K. (2013). Standardized nomenclature, symbols, and units for bone histomorphometry: a 2012 update of the report of the ASBMR Histomorphometry Nomenclature Committee. *Journal of Bone and Mineral Research*.

[24] Muschler G. F., Raut V. P., Patterson T. E., Wenke J. C., Hollinger J. O. (2010). The design and use of animal models for translational research in bone tissue engineering and regenerative medicine. *Tissue Engineering. Part B: Reviews*.

[25] Harding J., Roberts R. M., Mirochnitchenko O. (2013). Large animal models for stem cell therapy. *Stem Cell Research and Therapy*.

[26] Abrams K. L., Xu J., Nativelle-Serpentini C., Dabirshahsahebi S., Rogers M. B. (2004). An evolutionary and molecular analysis of Bmp2 expression. *The Journal of Biological Chemistry*.

[27] Reichert J. M., Rosensweig C. J., Faden L. B., Dewitz M. C. (2005). Monoclonal antibody successes in the clinic. *Nature Biotechnology*.

[28] Carragee E. J., Hurwitz E. L., Weiner B. K. (2011). A critical review of recombinant human bone morphogenetic protein-2 trials in spinal surgery: emerging safety concerns and lessons learned. *Spine Journal*.

[29] Schroff R. W., Foon K. A., Beatty S. M., Oldham R. K., Morgan A. C. (1985). Human anti-murine immunoglobulin responses in patients receiving monoclonal antibody therapy. *Cancer Research*.

