# Erratum to “Collagen Sponge Functionalized with Chimeric Anti-BMP-2 Monoclonal Antibody Mediates Repair of Critical-Size Mandibular Continuity Defects in a Nonhuman Primate Model”

**DOI:** 10.1155/2017/3689716

**Published:** 2017-12-03

**Authors:** Yilin Xie, Yingying Su, Seiko Min, Jianxia Tang, Bee Tin Goh, Leonardo Saigo, Sahar Ansari, Alireza Moshaverinia, Chunmei Zhang, Jinsong Wang, Yi Liu, Arash Khojasteh, Homayoun H. Zadeh, Songlin Wang

**Affiliations:** ^1^Molecular Laboratory for Gene Therapy and Tooth Regeneration, Beijing Key Laboratory of Tooth Regeneration and Function Reconstruction, Capital Medical University School of Stomatology, Tian Tan Xi Li No. 4, Beijing 100050, China; ^2^Laboratory of Tissue Regeneration and Immunology and Department of Periodontics, Beijing Key Laboratory of Tooth Regeneration and Function Reconstruction, Capital Medical University School of Stomatology, Tian Tan Xi Li No. 4, Beijing 100050, China; ^3^Laboratory for Immunoregulation and Tissue Engineering (LITE), Ostrow School of Dentistry, University of Southern California, Los Angeles, CA, USA; ^4^Department of Oral and Maxillofacial Surgery, Xiangya Stomatological Hospital, Central South University, Changsha, Hunan, China; ^5^Department of Oral & Maxillofacial Surgery, National Dental Centre, Singapore; ^6^Division of Growth and Development, School of Dentistry, University of California, Los Angeles, CA, USA; ^7^Division of Advanced Prosthodontics, School of Dentistry, University of California, Los Angeles, CA, USA; ^8^Department of Biochemistry and Molecular Biology, Capital Medical University School of Basic Medical Sciences, You An Men Wai Xi Tou Tiao No. 10, Beijing 100069, China; ^9^Department of Tissue Engineering, School of Advanced Technologies in Medicine, Shahid Beheshti University of Medical Sciences, Tehran, Iran

In the article titled “Collagen Sponge Functionalized with Chimeric Anti-BMP-2 Monoclonal Antibody Mediates Repair of Critical-Size Mandibular Continuity Defects in a Nonhuman Primate Model” [1], Dr. Songlin Wang should be listed as the second corresponding author.
