# Supplementary material for: Collagen Sponge Functionalized with Chimeric Anti-BMP-2 Monoclonal Antibody Mediates Repair of Critical-Size Mandibular Continuity Defects in a Nonhuman Primate Model
Source: Biomed Res Int. 2017 Mar 16;2017:8094152. doi: 10.1155/2017/8094152 (PMC5376406; doi:10.1155/2017/8094152)
Supplement: Supplementary file 1 — Supplemental Figure 1: 3D reconstructed CBCT images of a mandibular continuity defect at preoperative time point. 3D reconstructed images illustrate the landmarks used for 2D image analysis. Coronal (A) and sagittal (B) sections were obtained at equal distances from the anterior region to posterior region of defect as well as distance from buccal to lingual region of defect respectively. Supplemental Table 1: The bone density (HU) at three different zone within Axial, Coronal, and Sagittal places of CBCTs taken at 6 and 12 weeks. [file 8094152.f1.pdf]

Supplemental Figure 1

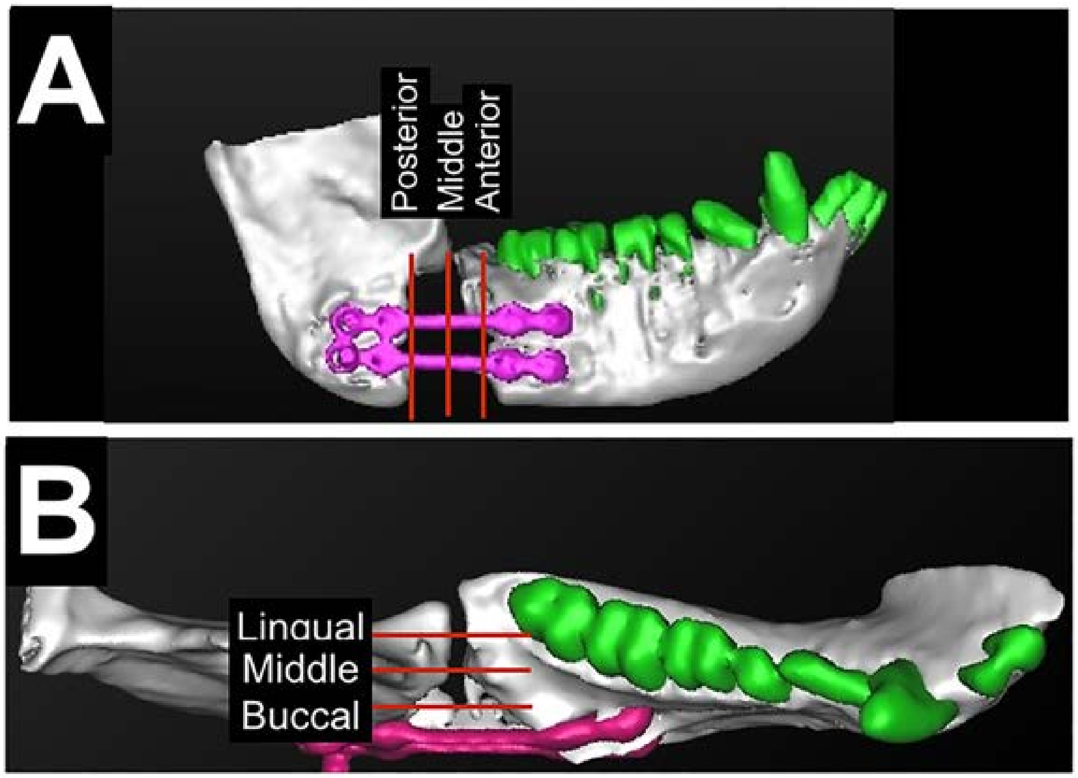

Supplemental data

Table 1. The bone density (HU) at three different zone within Axial, Coronal, and Sagittal places of CBCTs taken at 6 and 12 weeks.
Statistical significance: anti-BMP-2 mAb vs. Isotype mAb: *P<0.05, **P<0.01

1. Axial

	Superior	Middle	Inferior			
Pre-op	814±236	637±179	424±128			
	6 week			12 week		
	Superior	Middle	Inferior	Superior	Middle	Inferior
anti-BMP-2 mAb	106±117**	167±118**	145±75**	61±153	250±167**	150±126*
Isotype mAb	-119±110	-61±171	-137±94	-43±118	-73±171	-52±207

2. Coronal

	Anterior	Middle	Posterior			
Pre-op	726±111	727±184	889±190			
	6 week			12 week		
	Anterior	Middle	Posterior	Anterior	Middle	Posterior
anti-BMP-2 mAb	150±167	94±137*	137±201	122±226*	44±185	190±170
Isotype mAb	41±200	-33±110	-27±119	-67±134	-20±224	113±193

3. Sagittal

	Buccal	Middle	Lingual			
Pre-op	896±183	911±126	914±241			
	6 week			12 week		
	Buccal	Middle	Lingual	Buccal	Middle	Lingual
anti-BMP-2 mAb	48±112**	100±101**	92±109*	17±251	105±155	176±117*
Isotype mAb	-268±230	-138±133	-23±103	-103±116	-38±197	8±150
